# Functional resilience strategies for environmental destabilization of antagonistic yeast metabolite-mediated postharvest biocontrol management

**DOI:** 10.3389/fnut.2026.1930432

**Published:** 2026-10-22

**Authors:** Aasia Muhammed Jamiu, Fatimah Ahmed-Umar, Habiba Lawal, Gerefa Sefu Edo, Opoku Genevieve Fremah, Esa Abiso Godana, Frank Abimbola Ogundolie, Kaili Wang, Qiya Yang, Yingtong Zhang, Hongyin Zhang

**Affiliations:** 1 School of Food and Biological Engineering, Jiangsu University, Zhenjiang, Jiangsu, China; 2 Department of Biotechnology, Baze University Abuja, Abuja, Nigeria; 3 Institute of Agricultural Facilities and Equipment, Jiangsu Academy of Agricultural Sciences, Nanjing, Jiangsu, China

**Keywords:** antagonistic yeasts, environmental changes, food quality, functional resilience, postharvest biocontrol, postharvest nutrition

## Abstract

The biocontrol activity of antagonistic yeasts is through multiple mechanisms, one of which is metabolite-mediated antagonism involving volatile organic compounds, killer toxins, lytic enzymes, organic acids, and other bioactive compounds. However, the effectiveness of these yeasts in the management of postharvest diseases is affected by adverse environmental conditions such as preharvest climate drivers, postharvest storage microclimate, engineered preservation conditions, and supply-chain perturbations. These conditions may influence interactions between the yeasts, their metabolites, pathogens, and harvested products. Although some existing studies demonstrate that environmental conditions can affect yeast survival, colonization, metabolic activity, pathogen development, and postharvest nutritional quality, direct evidence simultaneously linking environmental stress, changes in yeast metabolite functionality, pathogen suppression, and preservation of nutritional value remains limited. This review therefore synthesizes evidence from various relevant studies to develop a functional resilience framework for yeast metabolite-mediated biocontrol. The framework takes resistance, recovery, persistence, stability, and functional redundancy as dimensions of resilience. The review also discusses stress preconditioning, protective delivery systems, responsive coatings, metabolite consortia, sensing technologies, and predictive approaches as potential strategies for promoting functional resilience. It also highlights integrated perturbation experiments, multi-omics, predictive validation, and assessment under postharvest storage and supply-chain conditions for future considerations.

## 1 Introduction

Postharvest disease management is entering a period in which biological control can no longer be evaluated only under stable storage conditions. With climate change, the atmospheric and physiological environments in which harvested commodities, spoilage fungi and biocontrol microorganisms interact are changing (1, 2). Higher temperatures, increased atmospheric CO_2_ during crop growing stage, changes in relative humidity and water activity during storage, disrupted supply chains and engineered preservation conditions may reshape postharvest disease ecology through accelerated fruit senescence, modified wound physiology, increased pathogen stress tolerance, and change in the distribution and aggressiveness of fungal pathogens (2, 3). These changes are especially important for species of *Penicillium*, *Botrytis*, *Alternaria*, *Aspergillus*, and *Fusarium*, whose growth, sporulation, toxin production, and virulence are strongly influenced by temperature, water activity, oxygen availability, CO₂, and host stress.

As postharvest losses already represent a major food security challenge (4, 5), environmental-associated destabilization of existing postharvest disease management systems may further compromise fruit quality, food safety, marketability, and sustainable supply chain resilience. However, the consequences of environmental-associated postharvest disease management failure extend beyond visible decay. Fungal colonization initiates extensive metabolic reprogramming within harvested tissues, accelerating oxidative stress, membrane degradation, enzymatic browning and respiration (6, 7). These processes collectively reduce the nutritional value of fruits and vegetables through depletion of vitamin C, phenolic compounds, flavonoids, anthocyanins, carotenoids, soluble sugars, and other bioactive constituents (8–10). Environmental fluctuations that weaken biological control may therefore indirectly compromise the nutritional quality delivered to consumers. Preserving yeast metabolite functionality under this stress should consequently be viewed not only as a disease management objective but also as a nutritional preservation strategy supporting food quality, consumer health, and sustainable food systems.

Some climate, storage and supply-chain environment variables co-regulate host physiology and microbial growth/infection dynamics (2, 3, 11, 12). These variables may interact in ways that can favor pathogen establishment, host susceptibility, and toxin risk. These drivers may also lead to pathogen redistribution or expansion to new geographic or commodity niches (13). Postharvest disease risk should therefore be seen as an emergent property of environment, host physiology, pathogen acclimation, and microbial community dynamics.

### 1.1 Why metabolite-mediated biocontrol matters

Antagonistic yeasts are some of the most promising sustainable alternatives to synthetic fungicides for postharvest disease control. These yeasts can colonize fruit wounds, compete for nutrients and space, and tolerate postharvest stress to ward of pathogens via multi mechanisms (7, 14, 15). This review focuses on the metabolite-mediated mechanism and how environmental destabilization can compromise it. Table 1 highlights these metabolites and other supporting mechanisms which include volatile organic compounds, killer toxins, siderophore-like iron-sequestering systems, lytic enzymes, oxidative metabolites, organic acids, and stress-responsive metabolites. This mechanism makes yeast biocontrol ecologically adaptable, but it also makes it vulnerable. Biological control may fail even when the yeast remains viable if associated stress destabilizes metabolite synthesis, secretion, diffusion, or persistence.

**Table 1:** Functional mechanisms of antagonistic yeasts in postharvest biocontrol and examples of representative factors.

Functional category	Role in functional resilience	Representative factors	Primary mechanism of action	Representative postharvest evidence (causality)	References
1. Direct secreted antifungal molecules	Primarily disease suppression.	Killer toxins (mycocins), volatile organic compounds, organic acids and, hydrolytic enzymes.	Membrane disruption, inhibition of spore germination, metabolic inhibition, and degradation of structural polysaccharides.	Suppression of *Penicillium expansum, Botrytis cinerea, Alternaria alternata, Colletotrichum* spp., on apples, grapes, citrus, peaches and tomatoes (mechanism associated with efficacy).	(113, 160, 175, 176)
2. Nutrient-competition factors	Competitive exclusion of pathogens.	Pulcherriminic acid / pulcherrimin iron uptake systems; nutrient scavenging.	Iron chelation, nutrient depletion.	*Metschnikowia pulcherrima* control of blue mold and grey mold (mechanism associated with efficacy).	(54, 55)
3. *Yeast self-protective and stress-response factors	Enhance stress survival, and recovery of antagonistic yeasts.	Trehalose, glycerol, mannitol, erythritol, heat-shock proteins and, antioxidant metabolites	Osmotic, oxidative, heat and desiccation protection.	Contributing indirectly to sustained disease suppression (mechanism associated with efficacy).	(44, 65, 166)
4. Host-defense and microenvironment-modifying factors.	Long-term colonization and reinforce host resistance,.	Biofilm extracellular polymeric substances (EPS), β-glucans, and mannoproteins.	Protective biofilms, occupation of wound sites, modification of the fruit microenvironment, and stimulation of host defense responses.	Enhanced colonization of fruit wounds and prolonged suppression of pathogens on apples, citrus, grapes and stone fruits (mechanism associated with efficacy).	(177, 178)

*These factors are not direct anti-fungal agents but support long-term biocontrol performance.

### 1.2 The Core problem: environmental stress destabilizes metabolite-mediated biocontrol systems

An antagonist yeast requires more than just surviving to be an effective biocontrol. Functional continuity between yeast stress physiology, metabolite biosynthesis, metabolite stability, pathogen susceptibility, and host-wound ecology are also required (16–18). Environmental-associated stress can disrupt this continuity at several mechanistic levels.

Firstly, elevated temperatures during storage may shift yeast transcription and metabolism away from antifungal production toward heat-shock response, membrane repair, antioxidant defense, and survival (12, 19, 20). This may also accelerate metabolite degradation, protein denaturation, VOC dissipation, oxidative modification, and enzyme inactivation (18, 21). Secondly, shifts in RH and water-activity may affect diffusion of metabolites (22). Lastly, pathogens exposed to these stress may develop enhanced virulence, and altered mycotoxin production (23–25).

Environmental-associated biocontrol failure should not just be interpreted as poor antagonist establishment. A yeast strain may colonize fruit wounds but produce the wrong VOC profile; it may remain viable but secrete fewer active enzymes; it may induce oxidative responses but quickly exhaust antioxidant capabilities; it may compete for nutrients but fail against a pathogen with enhanced efflux and detoxification capacity. This is why future postharvest biocontrol must be evaluated not only by lesion reduction under controlled conditions, but by functionality under fluctuating stress in different environments.

### 1.3 Review methodology

This is a narrative-based review supported by descriptive literature search. Relevant publications were primarily identified using Web of Science, Scopus, PubMed and Google Scholar using combinations of keywords including *yeast*, *biological control*, *postharvest disease*, *climate change*, *temperature*, *relative humidity*, *carbon dioxide*, *oxidative stress*, and *fruit storage*. Studies were selected based on their relevance to the major areas of the review, experimental evidence, postharvest applicability, and the availability of quality related outcomes. They also provided broad coverage of yeast species, commodities, pathogens, environmental stresses, and proposed mechanisms. Priority was given to recent studies where appropriate whereas others were used when they provided important mechanistic evidence. The selected studies therefore serve as representative examples of the available evidence rather than an exhaustive list of all published studies. In the description of evidence level, direct/indirect, limited and moderate are used qualitatively to indicate the strength and directness of available evidence rather than as formal quantitative evidence grades. These terms denote level of findings supported by multiple complementary studies or measurable biological outcomes and integration of the proposed causal sequence. Contextual evidence refers to component-level or related-system findings that provide mechanistic support but do not directly establish the complete yeast–pathogen–commodity relationship.

### 1.4 Defining functional resilience in postharvest yeast biocontrol systems

This review discusses postharvest yeast biocontrol functional resilience as the ability of yeast–metabolite–host systems to maintain stable production of antifungal metabolites, effectively suppress the establishment and development of pathogens, and preserve nutritional quality of products despite changes in environmental and storage-related stresses. Functional resilience is therefore a systems-level description of the interactions between the antagonist, its metabolite network, the host tissue environment, storage and supply-chain conditions.

The factors that determine resilience are physiological, ecological, and formulation-related properties which allow the antagonist to cope with environmental disturbances. On a microbial level, functional resilience means that antagonistic yeasts regulate stress responses, maintain metabolic activity, and redirect resources toward antifungal metabolite production rather than survival alone. Ecologically, resilience refers to the ability of antagonistic yeasts to preserve their competitive interactions within the changing postharvest microbiome. These determinants provide the underlying capacity for maintaining biocontrol activity but are not equivalent to successful disease control.

The components of functional resilience considered in this review as illustrated in Figure 1, are an emergent property of a system rather than a single physiological feature. They include resistance as the capacity to retain function during environmental, storage or supply-chain disturbance; recovery, as the ability to restore function after stress; stability, defined as maintained consistent performance under unfavorable conditions; persistence referring to the duration the biocontrol system continues to function above a predefined efficacy threshold during storage; and redundancy, as the ability to make up for functional losses through alternative mechanisms.

**Figure 1:**
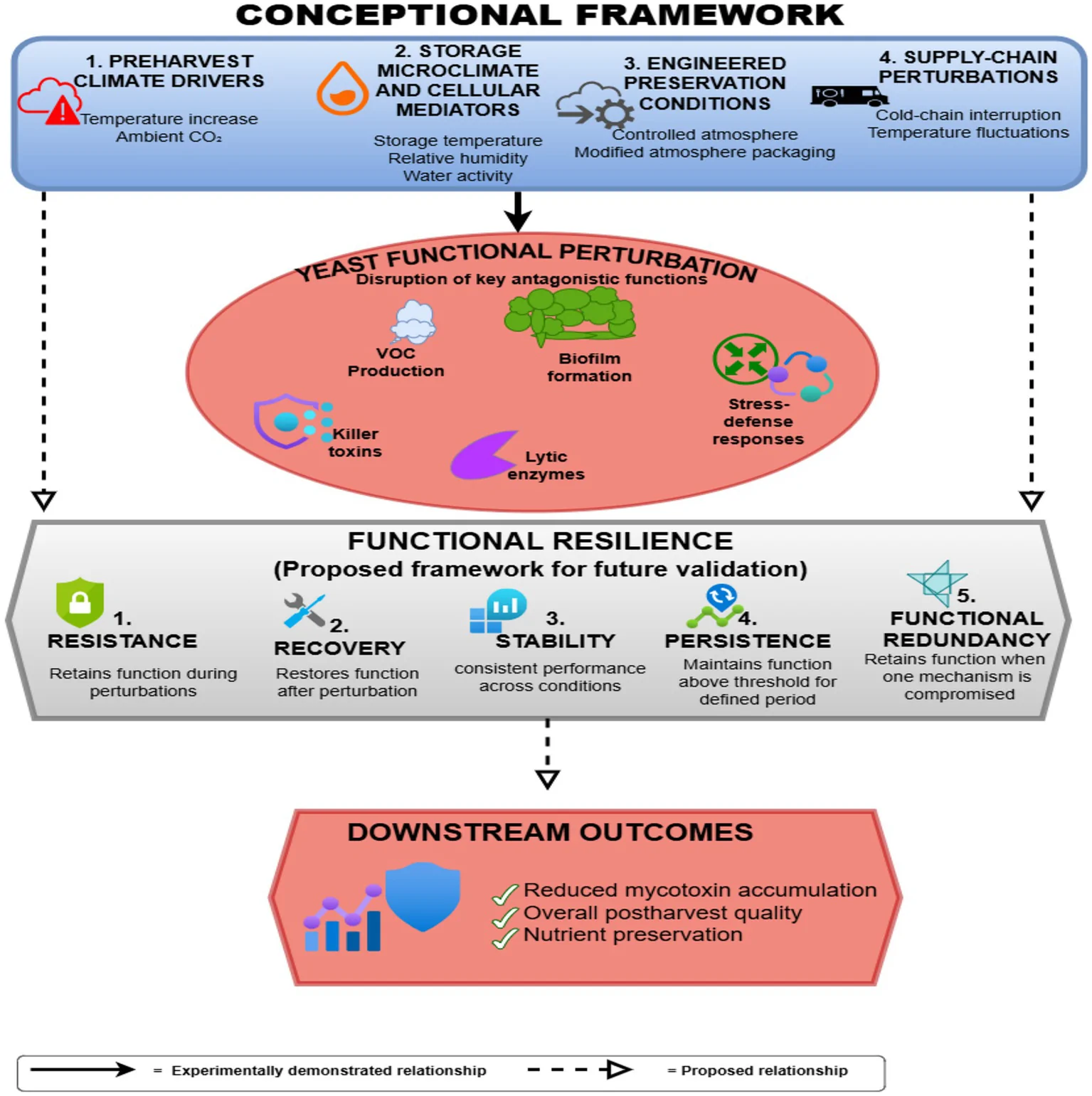
Conceptual framework illustrating the components of functional resilience in yeast-mediated postharvest biocontrol under environmental drivers occurring across different biological and management scales. Conceptual framework illustrating how environmental drivers operating at four distinct biological and management scales influence yeast functional resilience during postharvest storage: (1) preharvest climate drivers (2) storage microclimate (3) engineered preservation conditions and (4) supply-chain perturbations. Through different biological mechanisms, these drivers may disrupt key antagonistic functions. The framework considers resistance, stability, persistence, recovery and functional redundancy as attributes of the proposed functional resilience that may influence downstream outcomes.

Functional resilience is not indicated by a single outcome. Instead, it refers to the yeast metabolite-mediated biocontrol system’s ability to maintain or restore measurable antagonistic functions such as yeast viability and persistence, wound or surface colonization, antifungal metabolite abundance or activity, inhibition of pathogen growth, and lesion development or disease occurrence. These endpoints provide the primary basis for evaluating whether a biocontrol system remains functional under disturbances. Disease suppression and nutritional preservation are considered important downstream outcome of that retained functionality.

## 2 Drivers of functional destabilization in postharvest yeast mediated biocontrol systems

For this review, environmental stressors affecting postharvest yeast biocontrol are differentiated according to their source and the biological level on which they have an effect. Preharvest climate drivers may affect host physiology, pathogen pressure, and the existing microbiome before harvest. After harvest, storage microclimate has a direct effect on yeast metabolism and development of pathogens. Engineered preservation conditions are designed to alter the storage conditions while supply-chain perturbation may subject the harvested products to temporary environmental fluctuations during handling and distribution. All these variables work at various levels of time and management but the effects of each of them could affect the metabolic efficiency of yeast and thereby challenge functional resilience of yeast–metabolite–host systems.

Because there are few studies which have at the same time examined environmental stress, yeast metabolism, pathogen control, and host quality, this review draws on evidence from a number of related areas including postharvest biocontrol, yeast stress physiology, pathogen biology, food storage engineering, and food quality. Results from these studies were then analyzed and interpreted as conceptual links proposed through critical synthesis.

The subsequent sections describe environmental effects according to the endpoints directly measured in the original studies. In cases where the studies measured both environmental exposure and yeast physiological responses, metabolite activity, pathogen development, disease, toxin production, or quality outcomes, these relationships are stated as experimentally supported. However, when one or more links in the proposed sequence had not been measured directly, the relevant mechanistic relationship is described as inferred or hypothetical rather than established.

Table 2 gives representative examples of how different environmental conditions have been examined in postharvest yeast biocontrol systems. The examples also highlight important differences in evidential strength between direct postharvest experiments and contextual observations. Table 3 describes the sources, stage of occurrence, realistic exposure intensity, duration, and whether it represents a natural climatic factor, a supply-chain disturbance, a storage condition, or an active treatment.

**Table 2:** Representative examples of environmental and storage-associated factors influencing yeast biocontrol performance, with evidential context.

Stress type	Yeast strain/antagonist	Pathogen	Host commodity	Exposure range	Measured functional endpoint	Disease/toxin outcome	Evidence context	Reference
Elevated temperature (storage microclimate)	*Aureobasidium pullulans* (commercial BCAs)	*Botrytis cinerea*	Grape berries	15–30 °C	Biocontrol efficacy under different temperatures	Efficacy varied substantially.	Direct postharvest yeast–pathogen–host experiment.	(50)
High relative humidity (RH) (storage microclimate)	*Aureobasidium pullulans*	*Botrytis cinerea*	Grape berries	60–100% RH	Colonization efficiency and disease suppression	Strongly influenced biocontrol efficacy and pathogen control.	Direct postharvest yeast–pathogen–host experiment.	(50)
Cold storage temperature + RH	*Aureobasidium pullulans PL5*	*Penicillium expansum, Botrytis cinerea*	Apple, peach, plum	1–4 °C; 95% RH	Wound colonization, lytic enzyme production and nutrient competition.	Significant reductions in disease incidence.	Direct postharvest yeast–pathogen–host experiment.	(179)
* Controlled atmosphere (CA)/MAP storage (contextual evidence)	*Candida oleophila* (Nexy®)	*Penicillium expansum, Botrytis cinerea*	Apple, citrus, banana	Commercial cold storage (1–13 °C); CA/MAP environments.	Disease suppression	Demonstrated commercial efficacy under CA/MAP storage.	Partial/component or contextual evidence; CO₂ effect not directly tested.	(180)
Commercial storage variability	*Candida oleophila* (Aspire®, Nexy®)	*Penicillium expansum, Botrytis cinerea*	Apples, citrus, bananas	Commercial storage environments	Overall biocontrol performance.	Commercial efficacy is influenced by storage conditions.	Commercial validation; environmental effects inferred.	(181)

*Contextual commercial-storage evidence refers to studies conducted under commercial storage environments that demonstrate practical performance of antagonistic yeasts but do not directly test the effect of elevated CO₂ on yeast biocontrol efficacy through controlled atmosphere comparisons.

**Table 3:** Classification of environmental drivers affecting postharvest yeast biocontrol according to biological and management scale.

Environmental factor	Framework category	Where it occurs	Biological consequence
Gradual temperature increase (atmospheric warming)	Preharvest climate driver	Field	Alters fruit physiology, maturity, microbiome composition
Ambient atmospheric CO₂ enrichment	Preharvest climate driver	Field	Indirectly influences host physiology, fruit composition and microbial ecology.
Cold-chain and temperature fluctuations	supply-chain disturbance	Transport, distribution and retail	Influences yeast metabolism, respiration, pathogen development and metabolite stability.
Relative humidity	Storage microclimate	Storage	Surface hydration, condensation, and microbial colonization.
Controlled-atmosphere or Modified-atmosphere packaging	Engineered preservation condition	Controlled storage systems	Alters respiratory metabolism, redox balance and metabolite production under engineered conditions.
Water activity	Matrix property	Fruit wound/coating/media	Controls microbial growth and metabolism.
Oxidative stress	Cellular response	Yeast/pathogen/host	Causes redox imbalance and metabolic reprogramming

### 2.1 Preharvest climate drivers

#### 2.1.1 Rising temperature and gradual warming

Gradual increases in ambient temperature associated with climate change may influence postharvest disease management long before storage. Elevated growing temperatures may alter fruit physiology by accelerating maturation, modifying cuticle development, and influencing carbohydrate partitioning and antioxidant metabolism (26, 27). In addition, warming may affect pathogen distribution and inoculum pressure by altering the dispersal, survival, and epidemiology of fungal propagules (28, 29), while also reshaping the composition of epiphytic microbial communities colonizing fruit surfaces (30, 31).

Collectively, these preharvest changes may influence wound susceptibility to pathogens, nutrient availability, and the microbial communities; subsequently altering the performance of antagonistic yeasts during postharvest disease management. Rather than acting directly on postharvest biological control agents, these climate drivers may indirectly modify the host environment in which yeast-mediated antagonism subsequently occurs.

#### 2.1.2 Ambient atmospheric CO₂ enrichment

The physiological responses to gradual atmospheric CO₂ enrichment differ substantially from those observed under controlled-atmosphere storage. Elevated ambient CO₂ during crop growth may indirectly affect postharvest yeast biocontrol through alterations of crop physiology, tissue composition, pathogen ecology, and microbial interactions before harvest (32–34). These preharvest changes may then alter the ecological environment encountered by antagonistic yeast and pathogens during postharvest.

However, current evidence does not demonstrate that gradual atmospheric CO₂ enrichment directly alters wound-colonizing antagonistic yeasts in the same manner as percentage-level CO₂ concentrations used during controlled-atmosphere storage. Therefore, ambient atmospheric CO₂ should be regarded primarily as a preharvest climatic driver whose influence on yeast functional resilience is mediated through host physiological responses rather than direct effects on yeast metabolism.

### 2.2 Storage microclimate and cellular mediators

#### 2.2.1 Storage temperature

Storage temperature is among the most important environmental variables regulating postharvest microbial ecology. Appropriate refrigeration may slow fruit respiration, delay senescence, and suppresses pathogen development, whereas prolonged storage in higher temperatures may accelerate metabolic activity in both fruit tissues and microorganisms (35–37).

Temperature stress during postharvest storage, as one of the main driving forces for yeast metabolite destabilization may affect protein structure, membrane phase behavior, transcriptional regulation, secretion, volatility and degradation kinetics (2). Proteinaceous killer toxins and lytic enzymes are especially at risk due to dependence on correct folding, secretion, and extracellular stability (38). Heat stress may reduce this activity by causing partial protein unfolding, aggregation, and accelerated proteolysis (36). Thus thermally stressed yeasts may survive, but fail to deliver adequate extra-cellular enzyme activity at the infection interface, an example of which is the reduced efficacy of *Debaryomyces hansenii* against *Botrytis cinerea* on kiwifruit following heat stress (19).

Temperature stress also may lead to alterations in the function of organelles and protein secretion (39, 40). In response, the yeast may employ lipid remodeling, accumulation of trehalose, induction of heat-shock proteins, oxidation scavenging, glycerol metabolism and, stress responsive transcriptional programs (41–45). These responses however, come with a cost, as carbon, ATP and NADPH may then be diverted from antagonistic metabolite production to repair and survival (46). Although this trade-off has not been directly demonstrated for metabolite-mediated postharvest biocontrol under the corresponding storage conditions, a transcriptomic evidence shows that heat-stressed *D. hansenii* shifts toward stress-response regulation while losing biocontrol efficacy (18). In another study of native killer yeasts from citrus showed temperature-dependent protection, with some strains controlling *Penicillium* decay more effectively at 24 °C than at 7 °C (47).

Overall, temperature stress during postharvest storage may indirectly weaken antagonistic yeast biocontrol systems by disrupting protein stability, VOC behavior, membrane function, transcriptional allocation, and metabolite persistence.

#### 2.2.2 Relative humidity, free water, and water activity

Relative humidity (RH) represents an atmospheric property of the storage environment that strongly influences surface moisture and microbial colonization. Free water refers to liquid water present on or within the fruit surface or wound while water activity describes the thermodynamic availability of water within the fruit tissue, wound environment, edible coating, or culture medium.

##### 2.2.2.1 Relative humidity and free water

It has been hypothesized that the diffusion and local concentration of yeast metabolites within fruit wounds during storage may be influenced by RH and water availability (48) and that pathogen-derived extracellular polymeric substances may retain water, nutrients, or antagonistic compounds and potentially modify diffusion within the wound microenvironment (49). However, direct experimental evidence demonstrating that RH-driven formation of free water dilutes extracellular yeast metabolites or reduces their concentration at the yeast–pathogen interface remains limited.

The effect of high RH may be mediated partly through changes in surface moisture and the formation or persistence of free water. High RH environments enable moisture build up on fruit surfaces and wound sites, especially when temperature changes result in condensation. The thin water layers formed may then support the movement of extracellular enzymes and iron-chelating molecules, aiding microbial attachment and colonization. However, an increase in surface water can also favor pathogen germination, hyphal extension, and biofilm development (50), while free water at wound sites may also change how extracellular metabolites are distributed. It should be noted that these interactions remain largely hypothetical in fruit-wound systems.

##### 2.2.2.2 Water activity and metabolic availability

Water activity (aᵥ) represents the thermodynamic availability of water. Changes in aᵥ may influence microbial growth and metabolism even when visible free water is absent. At low aᵥ, yeast growth, diffusion of solutes, and extracellular secretion may be constrained. Desiccation can also destabilize proteins by disrupting hydration shells, increasing aggregation risk and potentially reducing enzyme flexibility (51). These effects could influence the production or activity of extracellular antagonistic factors, although their specific consequences for metabolite-mediated biocontrol in fruit wounds require direct validation.

Changes in water availability may alter substrate accessibility and the activity of competing microbial or host-derived enzymes, potentially modifying the effective contribution of yeast lytic enzymes to pathogen suppression (52, 53). Importantly, these effects should not be interpreted simply as consequences of high or low RH, because water activity within the wound or host matrix can differ from the atmospheric RH and can be affected by solute concentration, tissue composition, and other physicochemical properties.

Water availability may additionally influence metabolite-mediated competition for nutrients and minerals. For example, *Metschnikowia* species can suppress postharvest pathogens through pulcherriminic acid production, which chelates ferric iron and promotes the formation of insoluble pulcherrimin, thereby reducing iron availability to competing microorganisms (54–56). Changes in the state of hydration and physicochemical conditions of the wound may influence solute movement and iron distribution, but whether such changes directly weaken siderophore- or pulcherrimin-mediated antagonism has not been sufficiently demonstrated.

Overall, RH, free water, and water activity should be considered related but distinct determinants of the wound microenvironment. RH describes the surrounding atmospheric moisture, whereas free water represents liquid water that becomes available at the fruit surface or wound, particularly following condensation. Water activity describes the thermodynamic availability of water within the local matrix and can change independently of visible free water or atmospheric RH. Current evidence indicates that these moisture-related conditions can influence yeast growth, colonization, metabolic activity, pathogen development, and disease suppression, but direct evidence connecting specific changes in RH, free water, or water activity to altered extracellular metabolite concentration or activity at the yeast–pathogen interface remains limited. Future studies should therefore measure these parameters separately when evaluating moisture-dependent changes in yeast biocontrol performance.

#### 2.2.3 Stress conditions inducing oxidative stress

Oxidative stress is not an independent environmental driver but rather a physiological response induced by environmental perturbations including temperature fluctuations, altered gas composition, dehydration, and pathogen infection. Oxidative stress is hereby regarded as a downstream cellular response through which environmental disturbances influence functional resilience of antagonistic yeasts.

Oxidative stress may influence yeast biocontrol ability through a redox trade-off. ROS production can aid antagonism; as seen in some *Meyerozyma caribbica* strains that produce superoxide anions in fruit wounds, and oxidative signals may interact with host defense responses (57). However, excessive ROS damages yeast membranes, proteins, DNA, mitochondria, and secreted metabolites (58).

Under stress particularly high heat, exposure to excess light, increased UV radiation, cycles of dehydration rehydration, and fluctuations in atmospheric oxygen, ROS levels may exceed the yeast’s antioxidant capacity (3, 22, 59). In response, the yeast may reallocate NADPH, glutathione, catalase, peroxidase, and superoxide dismutase for self-protective action. This nay lead to reduced resources available for VOC biosynthesis, killer toxin secretion, lytic enzyme production, and iron-sequestering metabolism (58, 60). The activity of some secreted proteinaceous metabolites may also reduce through oxidative modification, including carbonylation, thiol disruption, fragmentation, or aggregation (61). Furthermore, VOCs may be oxidized, hydrolyzed, or photochemically transformed, changing their antifungal potency (62). In *Fusarium* species, temperature, water activity, and CO₂ interactively influenced growth and mycotoxin production (12). Furthermore, in storage CO₂ dissolves into aqueous films on fruit surfaces; hydration to carbonic acid and subsequent bicarbonate/proton equilibria can alter extracellular and intracellular pH (12, 63), and within microbial cells contributing to acid–base and membrane related stress (63).

Therefore, storage microclimate-associated stress may induce oxidative stress, convert a regulated antagonistic redox system into a chemically unstable environment where both yeast and metabolites deteriorate. Yeasts may survive through stress responsive pathways, but metabolite production becomes less reliable, while pathogens with antioxidant enzymes, and detoxification systems may become comparatively more resilient.

### 2.3 Engineered preservation conditions

#### 2.3.1 Controlled-atmosphere and modified-atmosphere packaging

Engineered Controlled-atmosphere (CA) storage and Modified-atmosphere packaging (MAP) conditions are different from gradual ambient atmospheric CO₂ enrichment. CA storage and Modified- MAP intentionally modifies oxygen and carbon dioxide concentrations to delay fruit respiration, reduce pathogen development, and extend storage life (64). Unlike ambient atmospheric CO₂ enrichment, CA and MAP exposes produce and associated microorganisms to substantially higher gas concentrations specifically designed for the purpose of preservation.

These engineered elevated CO₂ conditions may influence yeast respiratory metabolism, redox balance, volatile production, and metabolite secretion (65). This is hypothesized because in yeasts, maintenance of intracellular pH requires energy-dependent homeostatic processes, raising the possibility that ATP and reducing power could be redistributed from secondary metabolism toward stress maintenance (66). Whether such redistribution directly reduces antifungal metabolite production under CA and MAP conditions remains to be experimentally established.

### 2.4 Supply-chain pertubations

#### 2.4.1 Cold-chain interruption and temperature fluctuations

Following harvest, maintenance of an uninterrupted cold chain is essential for preserving both the quality of the produce and the efficacy of the biological control system. Cold-chain interruption and short-term exposure to elevated temperatures during transport, retail display, or temporary storage may increase fruit respiration, oxidative stress, alters membrane integrity, and create instability that may disrupt yeast colonization, metabolite production, and pathogen suppression. Because these disturbances occur during commercial handling over relatively short timescales rather than crop production, they represent supply-chain challenges rather than direct climatic drivers.

When repeated transitions between refrigeration and ambient temperatures occur, elevated temperature may raise vapor pressure and diffusion rates of antifungal VOCs. Studies have reported that such raise in vapor pressure can increase headspace concentration but also speed up volatilization loss from fruit wounds and packaging systems (67, 68). Warmer temperatures may convert sustained VOC inhibition into a short, unstable pulse and reshape VOC biosynthesis in a compound- and strain-specific manner (69, 70). This may result in a non-linear response where moderate warming may enhance emission, but higher temperatures reduce metabolite persistence and biosynthetic continuity. Repeated environmental fluctuations therefore represent an important commercial challenge because they may impair functional resilience without necessarily reducing yeast viability.

### 2.5 Pathogen stress acclimation and potential Long-term selection under environmental variability

Environmental stresses may induce rapid changes in pathogen responses. This includes transcriptional activation of stress-responsive genes, heat-shock proteins, antioxidant enzymes, and membrane remodeling pathways that enhance survival under adverse conditions (3, 71–73). However, these responses represent physiological acclimation rather than genetic adaptation, as they occur through regulatory and metabolic adjustments without requiring heritable changes. Nevertheless, repeated environmental disturbances may generate selective pressures that favor pre-existing stress-tolerant and new variants within pathogen populations, potentially altering population-level resilience over multiple generations (28).

Efflux pumps may be pivotal in such adaptations since ABC and MFS transporters have been associated with resistance to several chemical antifungal agents in postharvest pathogens. In *Botrytis cinerea*, multi resistance and virulence are linked to efflux transporters such as *atrB*, *mfsM2*, and *mfsG* (24, 74, 75). Similar transporter-based adaptation occurs in multidrug-resistant *Penicillium expansum* (76–78). However, these observations do not establish cross-tolerance to live antagonistic yeasts or to chemically diverse metabolites produced by yeasts. Whether transporter-mediated resistance to conventional fungicides also reduces susceptibility to yeast-derived VOCs, weak acids, phenolics, or other antagonistic metabolites therefore remains a testable hypothesis.

Nevertheless, it remains unclear whether repeated environmental selection can produce stable cross-tolerance to antagonistic metabolites produced by yeasts. Demonstrating such adaptation would require multigenerational selection under defined environmental conditions, followed by testing of independently maintained populations under common-garden conditions and direct challenge assays with the relevant yeast strains or purified metabolites. Summary Table 4 lays out the differences between pathogen pressures and environment-related adaptive mechanisms.

**Table 4:** Biological hierarchy of pathogen responses to environmental-associated stress and their potential implications for yeast biocontrol efficacy.

Biological response	Time scale	Genetic changes	Implications for yeast functional resilience	Representative examples	Current evidence
Immediate transcriptional responses	Minutes to hours	No heritable genetic change	May transiently modify susceptibility to antagonistic metabolites by activating stress-response and detoxification pathways.	Upregulation of oxidative stress genes, heat-shock proteins, catalases and detoxification enzymes following heat, oxidative or osmotic stress.	Indirect evidence only (41, 182)
Physiological acclimation	Hours to days	Reversible physiological/metabolic adjustment	May transiently increase pathogen tolerance to environmental and chemical stress, potentially reducing sensitivity to metabolite-mediated antagonism.	Increased membrane saturation, altered mitochondrial activity, increased antioxidant enzyme activity during prolonged heat or oxidative stress.	Moderate indirect evidence (87, 183, 184)
Phenotypic plasticity	Days to weeks	No heritable genetic change	May alter pathogen virulence and metabolite production under fluctuating environments, influencing disease outcomes.	Temperature- or humidity-dependent variation in patulin production, sporulation, melanin accumulation and virulence in *Penicillium expansum*, *Botrytis cinerea* and *Alternaria alternata*.	Moderate evidence (87, 182, 185, 186)
*Multi generational selection	Multiple generations	Primarily selection of standing genetic variation; new adaptive mutations may contribute over longer timescales.	May progressively enrich stress-tolerant pathogen populations through selection acting on standing variation and, over longer periods, adaptive mutations.	Repeated exposure to fluctuating storage environments favoring stress-tolerant isolates with improved survival or increased virulence.	Limited evidence (187)

* Proposed as a potential consequence of repeated environmental-associated stress.

Mycotoxin risk should be of particular concern because toxin production does not always follow fungal growth. Growth may be suppressed but temperature, water activity, and CO₂ may still allow toxin production, depending on species and environment (3, 12, 76, 79). In *P. expansum*, multidrug resistant phenotypes have been associated with increased patulin production, linking resistance physiology with food safety risk (76).

As illustrated in Figure 2, environmental destabilization may create a dual failure in the sense of weaker yeast metabolite function and more tolerant, virulent, or toxigenic pathogens.

**Figure 2:**
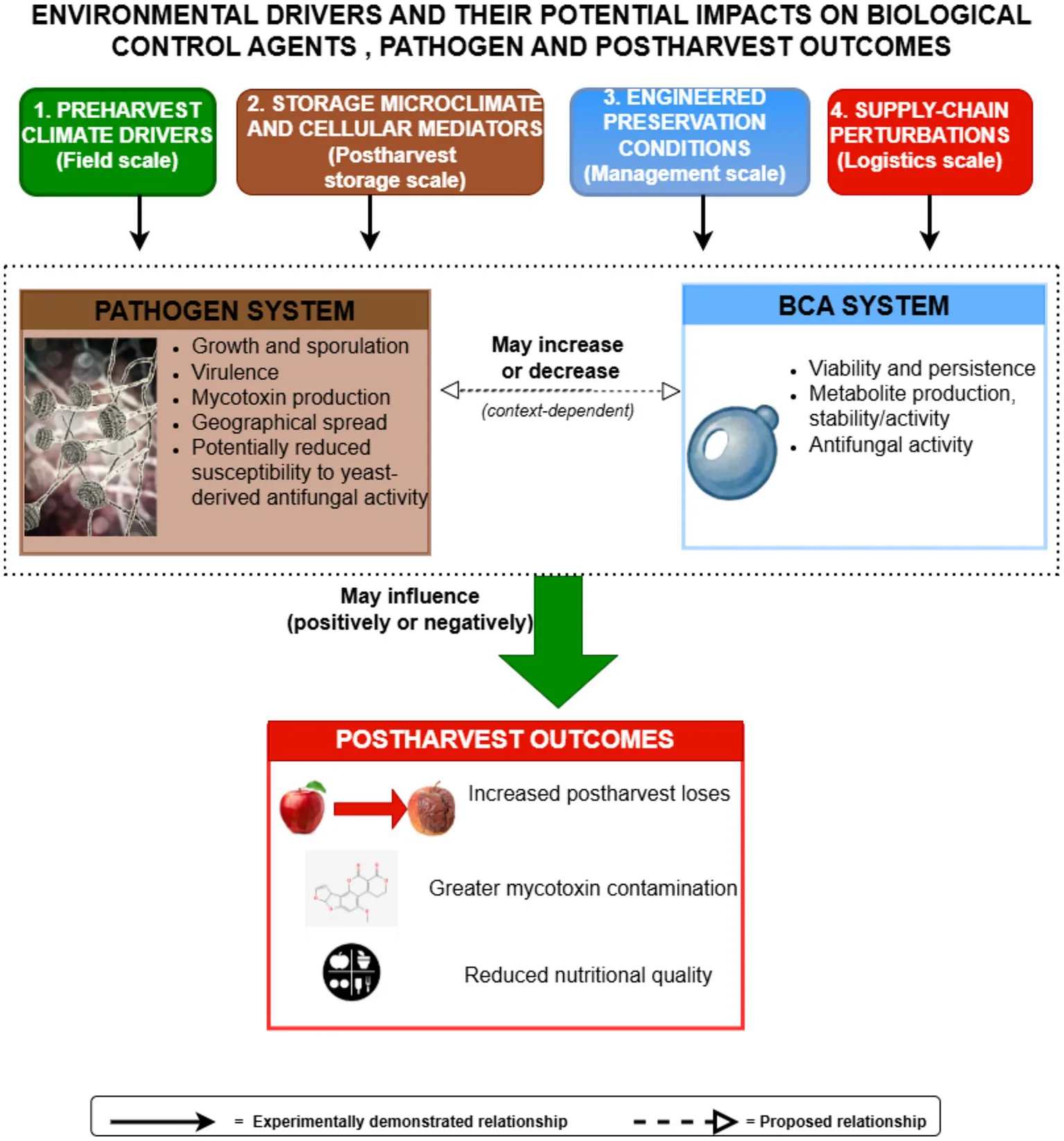
Environmental drivers and their potential impacts on biological control agent (BCA) performance, pathogen fitness, and postharvest outcomes. Environmental factors are organized according to four distinct biological and management scales: (1) preharvest climate drivers (2) storage microclimate (3) engineered preservation conditions and (4) Supply-chain perturbations. These environmental drivers may influence pathogen system, and BCA system. The direction and magnitude of these responses may depend on the specific environmental condition, exposure history, commodity, pathogen, antagonist, and whether the condition represents an environmental exposure or an intentionally engineered preservation treatment. Interactions between these systems may ultimately influence postharvest losses, food safety, and mycotoxin contamination.

### 2.6 Nutritional and bioactive compounds deterioration of fruits and vegetables

The implications of environment-associated disturbance of postharvest systems may go beyond the rise in decay incidences and the accumulation of mycotoxins. The loss of nutritional and bioactive compounds that define the functional quality of fruits and vegetables is another important implication. In typical storage conditions, harvested products remain metabolically active tissues, meaning that respiration, oxidative metabolism, and defense responses will continue. However, postharvest environmental conditions may upset this equilibrium and enhance biochemical deterioration.

Oxidative imbalance is what primarily directs the transition from the establishment of pathogen to the deterioration of nutrients (8). Healthy harvested fruits possess antioxidant systems involving ascorbic acid, phenolics, flavonoids, carotenoids, and enzymatic defenses to control ROS production. However, pathogen invasion may cause excessive formation of oxidants and metabolism consumes carbon resources and disrupts cellular compartmentalization in the host (35, 80). Therefore, when stress reduces yeast metabolite-mediated pathogen suppression, the resulting increase in fungal biomass may indirectly increase the depletion of nutritional antioxidants. However, the nutritional consequences discussed here are supported primarily by component-level evidence linking pathogen infection and environmental stress to biochemical deterioration, rather than by experiments simultaneously demonstrating environment-induced loss of yeast biocontrol function and subsequent nutrient depletion. The proposed connection between these processes therefore remains a systems-level hypothesis requiring direct validation.

#### 2.6.1 Ascorbic acid degradation, soluble sugar depletion and altered flavor quality under postharvest environmental stress

Because of its strong redox activity, ascorbic acid (Vitamin C) is one of the most important nutritional biomarkers affected by postharvest environmental instability. This vitamin serves as an important ROS scavenger in fruits and vegetables, maintaining cellular redox homeostasis and delaying deterioration (81, 82). However, this antioxidant function may also make it highly vulnerable to oxidation when ROS production exceeds its antioxidant regeneration capacity (10). Temperature variation, oxygen exposure, and accelerated respiration may increase conversion of vitamin C into dehydroascorbic acid and subsequently inactive degradation products (80, 83).

Various studies conducted on different fruit commodities show that both duration and temperature of storage have a great impact on retention of vitamin C content as its loss occurs in varying amounts depending on species, variety and the storage conditions (80, 84, 85). Such declines are attributed primarily to oxidation, increased respiratory activity, and stress-induced metabolic changes during storage.

Major determinants of sweetness, energy availability, and consumer acceptance of fresh produce like soluble sugars, including glucose, fructose, and sucrose, may also be primarily affected by oxidation reactions, while soluble carbohydrates may be directly utilized by postharvest fungal pathogens as carbon and energy sources (86). Therefore, failure of yeast-mediated biocontrol systems may result in a dual quality decline characterized by enhanced decay development and depletion of nutritional carbohydrates and vitamin C, leading to reduced sweetness, altered flavor profiles, and decreased market value of fresh commodities.

#### 2.6.2 Phenolic oxidation, PPO activation, and antioxidant reservoir depletion

Phenolic compounds are essential bioactive components of fruits and vegetables that add to total antioxidant activity while playing a role in the plant defense mechanisms (87).

During fungal infection, activation of polyphenol oxidase (PPO) and peroxidase (POD) leads to the oxidation of phenolic substrates into quinones which then polymerize into brown pigments responsible for tissue browning (88). Despite the fact that this response contributes to pathogen restriction through antimicrobial compound formation and barrier reinforcement, prolonged activation during uncontrolled infection may result in depletion of antioxidant phenolics and progressive quality deterioration (37).

Therefore, environment-associated weakening of yeast biocontrol may increase this transition from controlled defense activation to destructive oxidation. When yeast metabolites successfully suppress fungal germination and hyphal expansion, oxidative damage remains localized and manageable. But when pathogen accumulates, it may result in sustained enzymatic oxidation, membrane disruption, and depletion of phenolic reserves.

#### 2.6.3 Flavonoid and anthocyanin degradation during pathogen-induced oxidative stress

Flavonoids and anthocyanins contribute to the antioxidant potential, pigmentation, and consumer acceptance of many fruits such as berries. However, these compounds are highly sensitive to oxidative polymerization, enzymatic degradation, and environmental fluctuations (35). Under prolonged pathogen exposure, fungal-secreted enzymes, including glycosidases, oxidoreductases, and cell-wall degrading enzymes, can compromise cellular integrity and compartmentalization, facilitating the degradation and transformation of flavonoids and anthocyanins stored within vacuoles and other cellular structures (89, 90).

Anthocyanins are particularly vulnerable because their stability depends strongly on cellular pH, temperature, oxygen availability, and membrane integrity (85). Fungal colonization alter these through organic acid secretion and metabolic activity (91), then destabilization of anthocyanin structures and pigment loss may occur (92). The protective role of yeast biocontrol therefore extends beyond pathogen inhibition by maintaining the physiological conditions required for preservation of these bioactive compounds.

#### 2.6.4 Carotenoid oxidation and membrane deterioration

Carotenoids contribute nutritionally through antioxidant activity and provitamin A functions. However, their conjugated structures make them susceptible to oxidative cleavage (93). During pathogen infection, elevated ROS production from host defense responses and fungal metabolism can promote oxidative degradation of carotenoids, particularly when cellular membranes are disrupted (94). Because carotenoids are predominantly associated with plastid and membrane structures, pathogen induced lipid peroxidation can increase their susceptibility to oxidative attack and accelerate pigment loss and reduce their nutritional functionality (92). Temperature increase associated with storage environment variability may further accelerate carotenoid losses by increasing oxidation kinetics and metabolic activity during storage.

Postharvest environment variability may not always affect yeast biocontrol through isolated stress events. Rather, temperature, humidity, high levels of CO₂, and oxidative stress may work together to modify yeast–pathogen–host interactions. Therefore, while the combined stress may not suppress antagonistic yeasts, it may hamper their functional capacity by redirecting cellular resources from antagonistic metabolite production toward stress survival mechanisms. As a result, we can expect that weak antifungal activity and increased ability of pathogens to survive may lead to accelerated decay of the nutritional value of fruits and vegetables.

## 3 Maintaining functional resilience in yeast biocontrol systems

Although several commercial antagonistic yeast products have demonstrated the feasibility of microbial alternatives to synthetic fungicides, their performance remains strongly influenced by postharvest environment temperature, humidity, atmospheric composition, and pathogen pressure. Table 5 summarizes some of these commercial yeast-based biocontrol products.

**Table 5:** Commercial yeast-based biocontrol products: strains, regulatory status, and approved uses.

Commercial product	Active microorganism	Manufacturer/registrant	Jurisdiction	Approved use	Status and official source (accessed 14 September 2026)
Aspire®	*Candida oleophila I-182*	Ecogen Inc.	USA	Postharvest control of fungal decay on fruit.	Historical/discontinued (188)
Nexy™	*Candida oleophila* strain O	Agrauxine/Lesaffre; registration holders vary by jurisdiction	France/EU; USA	Postharvest biocontrol of fungal diseases on fruit; registered uses vary by jurisdiction.	Current (189, 190)
Botector®	*Aureobasidium pullulans* DSM 14940 & DSM 14941	SAN Agrow Holding GmbH/national registrants	Germany/EU.	Registered fungicidal use against fungal diseases including *Botrytis.*	Current in Germany (191)
Blossom Protect®	*Aureobasidium pullulans* (DSM 14940 & DSM 14941)	Registrant varies by jurisdiction	USA	Primarily preharvest disease management in pome fruits.	Current (192)
Candifruit™	*Candida sake CPA-1*	IRTA/Sipcam-Inagra	Spain/EU	Postharvest biological control of fungal decay on pome fruit.	Historical product; current status not verified
YieldPlus™	*Cryptococcus albidus*	Historical registrant/manufacturer	South Africa	Biological control of postharvest fungal decay on fruit.	Historical product; current status not verified
Noli® (formerly Shemer®)	*Metschnikowia fructicola* NRRL Y-27328	Koppert Biological Systems/local registrants.	Israel; France/EU	Preventive biological control of fungal diseases on fruit.	Current (193)

Regulatory status and approved uses are jurisdiction-specific and may change over time. “Current” indicates that an official regulatory or manufacturer source identified the product as registered/marketed in the specified jurisdiction at the time of access. “Historical product; current active registration not verified” indicates that the product is documented in the commercial or scientific literature but that a current official registration could not be confirmed. Product names, registrants, formulations, and approved uses may therefore differ among jurisdictions. Sources were accessed on 14 September 2026.

Four complementary approaches are particularly important. They are stress preconditioning of antagonistic yeasts, encapsulation and protective delivery systems, climate responsive edible coatings, and metabolite-consortia.

The resilience-oriented strategies discussed in this section differ substantially in their level of experimental validation. Stress preconditioning has been investigated directly in antagonistic-yeast systems, whereas other approaches, including responsive coatings, lipid-based carriers, nanoemulsions, microbial or metabolite consortia, sensing technologies and, artificial intelligence (AI), are supported by heterogeneous evidence ranging from postharvest formulation studies to related food, materials, sensing, and engineering systems. Accordingly, these approaches should not be considered equivalent to technological readiness.

### 3.1 Stress preconditioning of antagonistic yeasts

Stress preconditioning of antagonistic yeasts involves exposure of the yeast to a defined sublethal stress prior to application. This may cause the yeasts to mount protective transcriptional, metabolic, and structural responses that build adaptive resilience, for more challenging conditions in the fruit wound or storage environment.

For example, mild heat shock elicits induction of chaperones, endogenous trehalose accumulation, membrane stabilization, oxidative stress enzymes, and repair pathways (14) that render cells less susceptible to protein misfolding while protecting membrane integrity of cells and providing protective metabolic activity later in the context of thermal stress. In antagonistic yeasts, heat priming is not simply used for survival, it helps the antagonistic yeast to maintain persistence in the wound, respiratory competence and antifungal metabolite under shocks in storage temperature (18, 95). This has been proved in the improved stress tolerance and biocontrol performance in *Rhodotorula mucilaginosa* (96). Conversely, without appropriate adaptive priming heat stress can reduce biocontrol efficacy as shown in the work of (19). Controlled priming can therefore convert stress biology into adaptive preparation.

Osmotic conditioning describes another route of resilience. Postharvest fruit wounds are often accompanied by high concentration of sugar, low water activity and fluctuating osmotic pressure (6). While stresses such as heat and UV may further intensify oxidative pressure. Wang et al. (97) reported that sublethal exposure to salt in *Candida oleophila* induced antioxidant response and improved biocontrol efficacy, suggesting that osmotic conditioning may also cross-prime redox defenses in fruit wounds. Similarly mannitol and sorbitol pretreatments of *Debaryomyces hansenii* improved its antioxidant response and biocontrol performance (98). Compatible solutes therefore act as resilience stabilizers by protecting cells from osmotic and oxidative injury.

Sublethal oxidative stress can prime catalase, superoxide dismutase, glutathione-related pathways, thioredoxin systems, and repair mechanisms (99–101). This may help protect yeast cells against ROS damage to proteins and secreted enzymes. Zhang et al. (102) found that oxidative stress impaired viability and biocontrol efficiency of *Cryptococcus laurentii*, while glutathione supplementation improved oxidative tolerance and disease suppression. In another study, improved control over fruit rot pathogens were attributed to the sublethal oxidative acclimation of *D. hansenii*, leading to reduced ROS injury, improved wound growth, and allowed control of postharvest pathogens (18).

Transcriptomic memory may provide a basis for stress preconditioning. In yeasts, pre-stress exposure can affect the speed, magnitude, or duration of gene expression when stress is encountered later. This memory may involve epigenetic, transcriptional, protein, metabolic, and signaling changes. In postharvest biocontrol, transcriptomic memory could allow antagonistic yeasts to respond faster to climate driven stress. In the study of Wang et al. (97), Rlm1 mediated cell wall integrity signaling in *Candida oleophila* contributed to abiotic stress resistance and biocontrol efficacy. Recent studies also suggest that transcriptional regulators can link stress tolerance with postharvest performance (103, 104). Such transcriptional memory offers a strong mechanistic basis for selecting, priming, or formulating biocontrol agents (BCAs) according to their capacity for adaptive recall.

In mechanism, it can be said that stress preconditioning of the yeasts has potential to improve the ability of antagonistic yeast to retain biocontrol functionality under subsequent environmental stress by preserving redox homeostasis after application. The practical challenge is in calibration; priming must be strong enough to induce protective memory but mild enough to avoid metabolic exhaustion or suppression of antagonistic metabolite production.

### 3.2 Encapsulation and protective delivery systems

Encapsulation may support functional resilience by physically preventing yeast and their metabolites from adverse postharvest conditions. Under climate-associated stress, the failure points of most importance tend to be desiccation, heat injury, oxidative damage, rapid metabolite dissipation, and poor persistence at the wound site. Encapsulation within polymeric, lipid based, or emulsion based matrices may slow volatilization, improve local retention, and synchronize metabolite release with pathogen germination or early hyphal growth (105–107). More so, the protective matrices may help slowdown metabolite degradation and buffer exposure to the environment, possibly improving their efficacy and/or persistence of antifungal (108–110), depending on carrier composition and release behavior. In this way, encapsulation may convert a short-lived antifungal response into a more sustained delivery system.

Alginate encapsulation is one of the most widely studied because alginate forms biocompatible hydrogels upon cross-linking with calcium. For yeast biocontrol, alginate beads or microcapsules may provide a reservoir around the cells, attenuating dehydration, osmotic shock, and temperature fluctuation while allowing diffusion of VOC precursors, organic acids, enzymes and potentially signaling molecules (111).

For example, a study showed that *Saccharomyces cerevisiae* encapsulated in alginate beads remained viable while retaining functional properties after release (112), supporting the feasibility of alginate as a yeast delivery matrix. From a resilience standpoint, the alginate becomes a microhabitat that may attenuate stress and release yeast cells without causing shock through abrupt exposure. This technique can be applicable to volatile and hydrophobic antifungal compounds. Yeast VOCs and related low molecular weight metabolites, like fatty acids and esters are difficult to keep at effective concentrations as they volatilize and oxidize and can be partitioned into the waxes of the fruit surface or dissipate through packaging exchange (113).

Liposomes and lipid-based carriers can attract particularly hydrophobic and membrane-active metabolites. Their phospholipid bilayer allows the incorporation of hydrophobic compounds, aids dispersibility in aqueous coatings, reduces exposure to oxidation or photocatalytic degradation, and allows for controlled release via diffusion, membrane destabilization, and reactivity to environmental conditions (108, 114, 115). Lipid-based carriers may improve the protection, stability, or delivery of yeast-derived antagonistic compounds; however, their ability to maintain yeast-mediated biocontrol function under environmental perturbations remains to be established.

Nanoemulsions may allow scaled-up, high-dispersion of lipophilic antifungal compounds and low-water-solubility metabolites. Small droplet size aids dispersion, increases interfacial area, apparent stability, and allows for controlled release (116, 117).

In yeast biocontrol systems, nanoemulsions may be utilized as protective co-formulants that stabilize yeast-compatible antifungal compounds, or as hybrid systems where colonization is provided by the yeasts, with the nanoemulsion providing chemical pressure in the critical early periods. This dual-phase design has potential to improve the stability and delivery of bioactive antimicrobial components.

### 3.3 Climate-responsive edible coatings

Traditional edible coatings slow down moisture loss and respiration rates, modify gas exchange, and carry antimicrobial compounds of biocontrol agents. A resilient edible coating should sense or respond to microclimatic stress and rapidly release some biological or metabolic protection when the risk of pathogen establishment increases. Such edible coating could feature three layers of resilience: physical buffering, biological delivery, and a triggered, localized release of metabolites. The physical layer could reduce water loss, modifies gas exchange and oxidative exposure (118, 119). The biological layer could keep the yeast viable and enhance their local colonization (120, 121). The trigger layer responds to changes in humidity, temperature, CO2, pH, or enzymes released from the invading pathogen (122–124).

As stated in the study of (23) that pathogen growth will often cause acidification of the wound microenvironment, a coating triggered by pH change could release some antifungal metabolite in response. It could also be the case that some pathogen-secreted pectinases, cutinases, etc. could act as enzymatic triggers for coating degradation and metabolite release (125). This would transform edible coatings from static preservation barriers into adaptive biocontrol regulators.

Humidity-triggered release merits attention since high relative humidity and free surface moisture can induce fungal germination, whereas low humidity can desiccate the yeast cells and reduce metabolite diffusion (126). A smart coating could be designed to swell in response to higher humidity, forming larger pores and releasing yeast metabolites, when disease germination risk peaks. Under lower humidity, the same matrix could be more compact and inadvertently retain moisture around yeast cells to avoid premature loss of metabolite. This may provide a protective delivery strategy by time-correlating antifungal release to infection pressure rather than over-dosing antimicrobial metabolites. The development of dual-responsive cinnamaldehyde nanofiber films is an example of such; antimicrobial compounds are released from the films in response to a relative humidity increase and a pathogen induced decline in pH (123).

Temperature-responsive systems could release antifungal compounds during temperature fluctuations or slowly release and preserve metabolites in cold storage until temperature abuse occurs during transport or retail handling. This design approach can be found in thermo-responsive hydrogels and nanocarriers (127, 128). Their application to yeast biocontrol is underdeveloped, but the mechanistic rationale is strong.

Studies supporting coatings as metabolite retention platforms include Kharchoufi et al. (121) demonstrated that chitosan or locust bean gum coatings incorporating pomegranate peel extract and *Wickerhamomyces anomalus* reduced *Penicillium digitatum* decay on oranges and González-Estrada et al. (129) used arabinoxylan coatings entrapping *Debaryomyces hansenii* to control blue mold decay on Persian lime.

Instead of edible coating focusing only on improving shelf life under one storage condition, future studies should test whether the coating retains yeast metabolite function across fluctuating climate and postharvest conditions.

### 3.4 Metabolite consortia and systems-level biocontrol

Metabolite consortia may help with maintaining resilience by increasing ecological redundancy. A clonal yeast often utilizes a restricted arsenal of antagonistic actions (4, 14, 130), and one or more of these functions may fail under stress. Combining complementary antagonistic metabolites or biocontrol agents may provide functional redundancy and potentially improve robustness under variable environmental conditions. However, the ability of such consortia to maintain metabolite-mediated antagonism across defined perturbation regimes requires direct experimental validation.

Ecological redundancy is not repetition; it is insurance for functionality. In a resilient consortium, different strains may contribute complementary functions such as colonization, and biofilm stabilization. If climate or postharvest environment-associated stress hinders one function, the remaining functions should still provide partial disease control. This is similar to distributed robustness in ecological systems, where diversity increases stability under environmental pressure (131, 132). For postharvest biocontrol, target should be metabolically complimentary strains, equipped with optimal combinations of growth kinetics, non-antagonistic interactions, and different responses to stresses.

Cooperative metabolites are central to this concept. VOCs may inhibit early spore germination (133), creating time for yeast colonization. Lytic enzymes may weaken the pathogen cell wall, increasing sensitivity to killer toxins or redox stress. Siderophore-mediated iron depletion may reduce pathogen respiration and virulence (134), while redox-active metabolites and host-induced defenses may increase oxidative pressure (15). Biofilm-forming yeasts can stabilize the antagonist population on wounds and retain metabolites locally (49). These interactions may produce additive or synergistic suppression, especially when pathogen pressure is high or there is fluctuation in the storage or transport environment.

Existing studies lend support to the feasibility of mixed biological systems. Kefir-derived yeast–bacterial consortia inhibited *Penicillium expansum* on apple resulting in increased biocontrol activity (135). Against diverse fungal rots on citrus, yeast mixtures were also used (131). These findings support the concept that resilience can be achieved from consortia function.

An advanced path will be Microbiome-informed consortia. Fruit surfaces contain native microbial communities shaped by cultivar, orchard, geography, maturity, storage, fungicide history, and stress (136, 137). Instead of importing a single antagonist into an unfamiliar microbial network, microbiome-informed design can help identify core beneficial taxa that are already adapted to the fruit surface and combine them into synthetic consortia. This approach could better establishment, reduce incompatibility, and enhance resilience under climate or postharvest environment conditions.

#### 3.4.1 Challenges of metabolite consortia

A consortium may pose risks that must be addressed. Coexisting yeast strains may compete for carbon sources, attachment sites, and micronutrients within fruit wounds (138, 139) resulting in dominance of fast growing but less antagonistic populations. Also, some strains may produce inhibitory compounds, that negatively affect partner strains and alter the overall metabolic balance of the consortium (131). Such interactions may reshape VOC profiles and secondary metabolite production, potentially reducing the synergistic antifungal activity expected from consortium-based systems.

Another major challenge is the fluctuation of population abundance during storage. Differences in growth kinetics, stress tolerance, nutrient utilization strategies, and environmental adaptability may cause progressive shifts in consortium composition, resulting in loss of the intended functional balance (140–142).

Future studies may overcome these limitations by incorporating microbiome-guided strain matching. Candidate yeast combinations can be selected based on ecological relationships within the native fruit microbiome, metabolic compatibility, and predicted cooperative interactions. Integrating metagenomics, metabolomics, and network-based microbial ecology approaches can help identify complementary strains that can occupy distinct ecological niches while minimizing competitiveness.

A useful consortium should be evaluated based on functional stability under climate- associated and storage environment variabilities as well as pathogen acclimation. Key performance metrics should include metabolite redundancy, enzyme activity, pathogen stress-marker expression, and mycotoxin suppression.

## 4 Smart monitoring technologies for tracking postharvest quality and functional resilience

Smart technologies such as hyperspectral imaging, electronic noses, and biosensors are regarded as monitoring and diagnostic technologies rather than direct resilience-maintaining interventions.

Existing studies have successfully applied HSI for early detection of fungal infection, mapping of decay development, bruising identification, firmness estimation, maturity evaluation, and prediction of storage-related quality changes (143–145).

Similarly, electronic nose systems have been investigated for monitoring volatile fingerprints associated with fruit ripening, microbial spoilage, and fungal infection (146, 147). By detecting complex volatile patterns generated during storage, e-nose technologies can distinguish healthy commodities from deteriorating products and provide early warnings of spoilage development.

Artificial intelligence may support monitoring or prediction of resilience-related changes by improving classification accuracy, pattern recognition, and predictive modelling of quality parameters (148).

These tools can detect spectral, volatile, chemical, or environmental signatures associated with pathogen development, commodity deterioration, or storage conditions, but such signals do not necessarily represent the functional state of the antagonistic yeast. Their application to resilience assessment therefore requires calibration against measurements such as yeast viability, persistence, metabolite abundance or activity, pathogen growth, lesion development, or other defined functional endpoints. Once validated, these measurements could support estimation of the current postharvest system state and identification of conditions associated with declining biocontrol performance.

### 4.1 Plausible transferability: integrating multimodal datasets to predict functional resilience

Currently, these technologies individually provide fragmented information: hyperspectral imaging captures changes in fruit biochemical composition, electronic noses detect volatile alterations, metabolomics identifies chemical responses, molecular tools characterize microbial populations, and AI models recognize complex patterns within large datasets. The next stage in smart postharvest monitoring should involve combining the above established technologies with microbial, metabolomic, and environmental information to develop predictive indicators of antagonistic yeast functional resilience.

#### 4.1.1 Hyperspectral and multispectral imaging

Hyperspectral and multispectral imaging have the potential to support more robust biocontrol resilience through early detection of instability in the postharvest yeast pathogen system before disease becomes manifested visually. Usually, inspection detects decay only after pathogen colonization, tissue browning, or sporulation has progressed (149–151). By that stage, yeast antagonists may already have failed to colonize the wound or produce sufficient metabolites to suppress pathogen germination.

Mechanistically, fruit quality and nutritional properties are altered by fungal infection. Because many of these biochemical changes occur during the early stages of pathogen invasion and environmental stress, such spectral changes may be captured within narrow bands by hyperspectral imaging, or fewer bands by multispectral imaging in industrial settings (152). Reflectance alterations in visible and near-infrared reflectance patterns may then be associated with changes in important quality and nutritional parameter, including vitamin C, phenolic compounds, anthocyanins, soluble solids content, firmness, pigment composition, and oxidation-related tissue deterioration (153–155).

In this sense, imaging may act as a spatial decision tool that can identify the precise location and intensity of biocontrol failure. Instead of applying yeast antagonists or metabolites uniformly across an entire fruit lot, spectral imaging can guide localized treatment.

#### 4.1.2 Biosensors and real-time monitoring

Biosensors and real-time monitoring systems may provide indicators associated with biocontrol performance by monitoring the dynamic storage variables that determine whether the yeast metabolites remain active.

So far, electrochemical and optical biosensors have shown considerable promise for rapid, non-destructive monitoring of key postharvest quality indicators, including ascorbic acid, phenolic compounds, antioxidant capacity, sugars, and pathogen-associated volatile compounds (156, 157). Integrating these sensors with environmental monitoring systems could allow simultaneous assessment of storage conditions, microbial activity, yeast metabolic competence, and nutritional stability within a single monitoring framework.

Pathogen prediction may be enhanced through VOC-based sensors and electronic noses. Spoilage fungi and infected fruit tissues have specific emitted VOCs signature pattern (146, 147). The electronic nose systems do not need to identify all the volatile molecules but detect the volatile fingerprint associated with either early infection, pathogen species or the stage of spoilage.

Conceptually, biosensors may also be used to monitor metabolite activity directly. For example, if yeast-associated VOCs decline as pathogen-associated VOCs increase, that could suggest loss of volatile-mediated suppression. A low local redox-buffering capacity could mean oxidative stress depletes the antagonist (158). Increased free iron could indicate weakening of siderophore-mediated competition (159).

These signals would mean biocontrol is no longer a one-time biological treatment, but a living functional system to be managed. The key advantage is timing; interventions are most effective before pathogens pass the germination and early colonization threshold. Once visible rot develops, metabolite reinforcement is often too late.

#### 4.1.3 Artificial intelligence for monitoring, prediction, and decision support

Artificial intelligence should not be framed simply as a trend-driven analytical tool. Its potential value in postharvest biocontrol lies in integrating environmental, microbial, metabolic, and quality-related measurements to support state estimation, failure prediction, and, potentially, closed-loop intervention. The latter requires substantially stronger validation than monitoring or prediction alone.

State estimation refers to determining the current condition of the commodity, antagonist, pathogen, or storage environment from measured data. Disease risk and biocontrol performance are influenced by interacting variables, including fruit cultivar., maturity, wound load, pathogen inoculum, antagonist physiological state, temperature history, RH, condensation, CO₂/O₂ balance, metabolite release kinetics, and previous stress exposure (2, 59, 160, 161). Machine-learning approaches could integrate spectral images, biosensor outputs, environmental measurements, and historical storage records to characterize current storage and biological conditions. Such approaches may also help distinguish whether observed risk is associated with heat stress, RH instability, gas imbalance, declining yeast activity, pathogen activity, coating deterioration, or fruit senescence (5, 162). However, sensor-derived signals should not automatically be interpreted as direct measures of yeast-mediated biocontrol function and require appropriate biological ground-truth measurements.

Failure prediction represents a higher level of application in which integrated measurements are used to estimate the probability or timing of declining biocontrol performance or postharvest deterioration. By learning relationships among environmental, microbial, and quality-related variables, AI-based models may support prediction of conditions associated with declining yeast biocontrol performance. Machine-learning algorithms could also be used to estimate disease progression and changes in quality-related parameters, including vitamin degradation, phenolic oxidation, sugar depletion, and antioxidant capacity, under dynamic storage conditions. However, these predictions should distinguish correlation with quality deterioration from evidence that a specific decline in yeast-mediated function preceded or contributed to that deterioration.

Failure prediction may also be particularly relevant to the timing of metabolite-mediated antagonism. Different yeast antagonistic factors may act at different stages of pathogen development. For example, VOC-mediated activity may be most relevant before or during spore germination, lytic enzymes require contact with developing hyphae, and competition for iron may be more effective before substantial pathogen biomass has accumulated. Redox-mediated effects may also depend on maintaining an appropriate oxidative environment without imposing excessive stress on the antagonist (163).

Environmental variability may alter the timing of pathogen germination and yeast metabolite production, creating an opportunity for models to estimate periods of increased disease risk or reduced antagonistic activity. Nevertheless, such applications remain prospective unless the predicted biological states and timing relationships are experimentally validated.

Closed-loop intervention represents a higher level of system integration, in which model outputs trigger or regulate a defined intervention. For example, if a validated model identifies high RH and condensation as conditions associated with increasing disease risk, the system could potentially trigger modification of humidity management or adjustment of a protective coating. However, this represents proposed applications rather than established capabilities.

State estimation and failure prediction represent more immediate applications, whereas closed-loop intervention requires substantially stronger experimental and operational validation. AI should consequently be viewed as a potential decision-support framework for evaluating and managing functional resilience rather than as an intervention that inherently maintains resilience.

## 5 Future research directions

Coordinated advances in multi-omics, synthetic biology, predictive microbial ecology, and translational validation can help in moving research beyond identifying antagonistic yeast strains toward designing biocontrol systems that retain functionality under postharvest environment variability.

### 5.1 Multiple omics (multi-omics) for resilient biocontrol

In identifying molecular traits that predict stable biocontrol multi-omics can be used. Transcriptomics, proteomics, and secretomics can link stress regulation with active antifungal proteins, transporters, and antioxidant systems (48, 164, 165). Metabolomics should quantify metabolite abundance as well as release kinetics, persistence, spatial distribution, and activity under changing conditions during storage or supply chain.

Biomarkers such as inhibitory VOC thresholds, antioxidant capacity, lytic-enzyme activity, metabolite ratios, and iron-depletion signatures could support rapid strain screening, formulation design, stress-preconditioning protocols, and real-time monitoring. Systems-biology models should then integrate environmental inputs, yeast physiology, pathogen acclimation, host responses, and disease outcomes to identify the combinations of traits that predict reliable control.

### 5.2 Synthetic biology and engineered yeasts

Synthetic biology offers a way to help improve the predictability and environmental responsiveness of yeast biocontrol. Two pathways are relevant: engineered living antagonists where acceptable, and engineered yeasts as production platforms for standardized antifungal metabolites.

Advanced designs could couple metabolite production to environmental or pathogen-associated signals. Stress-responsive promoters could activate antifungal output during environmental stress or pathogen activity. Candidate pathways include trehalose (166) and glycerol metabolism, glutathione-dependent redox regulation, heat-shock responses, membrane lipid remodeling, DNA repair, and cell-wall integrity (19, 42, 167, 168). However, enhanced survival should not be treated as sufficient evidence of improved biocontrol. Engineered strains must retain wound-colonization ability, metabolite output, ecological competitiveness, and sensory neutrality under climate or postharvest environment-associated stress.

Regulatory requirements will be particularly demanding for engineered yeasts, microbial consortia, and metabolite-responsive systems. A live antagonistic yeast intended to suppress a postharvest pathogen through a plant-protection claim may require assessment of the microorganism as an active biological agent together with the formulated product, its efficacy, exposure, residues where relevant, environmental fate, and effects on non-target organisms. In the European Union, plant-protection products and their active substances are governed by the plant-protection regulatory framework established under Regulation (EC) No 1107/2009, which addresses the approval of active substances and the authorization and placing on the market of plant-protection (169). In the United States, microbial pesticides are regulated within the Environmental Protection Agency’s biopesticide registration framework, which includes specific requirements for microbial pesticide products (170). By contrast, a purified yeast-derived metabolite intended for direct food use may be subject to food-ingredient or food-additive requirements depending on its composition and intended conditions of use (171). A metabolite or microbial preparation making a pesticidal claim may instead fall within a pesticide-specific regulatory framework. Similarly, coatings and other materials intended to contact food are subject to food-contact-material requirements, including considerations of migration, intended use, food composition, and organoleptic properties (172). Thus, the applicable regulatory pathway depends on the nature of the biological or chemical agent, its formulation, intended use, and the regulatory claim made for the product.

For synthetic and engineered yeast approach, safety evaluation should include genetic stability, antimicrobial-resistance potential, unintended metabolite production, environmental persistence, non-target effects, and exposure. Development programs should distinguish early between products intended to contain live organisms and those based on purified metabolites or non-viable derivatives, because their regulatory and safety assessment pathways may differ substantially.

### 5.3 Artificial intelligence driven predictive microbial ecology

For AI based monitoring and prediction to provide reliable decision support, models should be trained and evaluated against appropriate measurements and subsequently tested using independent datasets. External validation across different commodities, storage facilities, environmental conditions, and production settings is particularly important because relationships between sensor signals and biological outcomes may vary across systems (173). Model uncertainty and prediction errors should also be quantified, while actionable thresholds should be established before predictions are used to guide postharvest interventions. These requirements are especially important for distinguishing measurements that merely correlate with deterioration from indicators that reliably reflect changes in yeast metabolite-mediated biocontrol function.

The integration of complementary environmental, microbial, metabolic, and nutritional datasets could therefore support future prediction of biocontrol performance under variable storage conditions. Digital twins represent a prospective framework for such integration. Such models may eventually help characterize functional resilience through measurable variables such as retained pathogen suppression, metabolite stability, yeast persistence, and nutritional preservation. However, this will require validation through controlled environmental and storage stress trials.

Therefore, the future direction should be AI-assisted adaptive biocontrol models that predict which strain, formulation, coating, atmosphere, and support treatment should be used under specific environment or storage risk conditions. The strongest approach will combine mechanistic models with machine learning (148, 174).

### 5.4 Industrial translation challenges and future perspectives

Translation should be incorporated into research design from the beginning rather than treated as a final commercialization step. Promising biocontrol systems frequently fail because laboratory efficacy does not translate across cultivars, pathogen populations, storage atmospheres, packinghouse practices, and cold-chain disruptions. Future studies should therefore prioritize commercial-scale validation under variable environmental conditions.

Standardized testing frameworks are also important. Studies should report strain identity, formulation composition, application dose, cell viability, metabolite activity, storage stability, sensory effects, treatment cost, and performance during realistic temperature and humidity fluctuations. Trials should include multiple cultivars, mixed pathogen populations, native microbiota, and supply-chain perturbations. Resilience claims should be based on retained function across these conditions rather than on maximum efficacy under a single optimized environment.

Economic feasibility is equally important. Fermentation, drying, encapsulation, smart coatings, biosensors, and AI infrastructure add cost and operational complexity. Techno-economic analysis should therefore accompany biological optimization. A successful product must reduce losses or chemical input sufficiently to justify production, monitoring, training, and equipment requirements.

Finally, research should therefore prioritize systems that are biologically robust, operationally simple, economically justified, and compatible with regulatory and consumer expectations.

## 6 Conclusion

Advances in biocontrol approach for management of postharvest diseases will depend on resilient biocontrol systems capable of maintaining antifungal functionality under increasingly variable postharvest environmental conditions. Its reliability will depend not only on the presence of an antagonistic yeast, but also on sustained metabolite biosynthesis, stability, and pathogen target susceptibility. This reliability may be destabilized by unfavorable environmental exposures including preharvest climate drivers, supply-chain perturbations, storage microclimate, and engineered preservation conditions.

Therefore, the challenge for future postharvest disease management is not only to discover more effective antagonistic yeasts, but to preserve the function of their antifungal metabolite networks. This objective extends beyond preventing produce decay to maintaining the biochemical integrity of harvested fruits and vegetables. Creating resilient yeast–metabolite–host–environment systems that are adaptive, measurable, and operationally robust across variable conditions will be key to maintaining or restoring these antifungal functionalities. Complementary approaches to consider in achieving these include stress-preconditioning, protective delivery matrices, climate-responsive edible coatings, metabolite consortia, biosensors, spectral imaging, and AI-driven predictive platforms.
